# Next generation immuno-oncology tumor profiling using a rapid, non-invasive, computational biophysics biomarker in early-stage breast cancer

**DOI:** 10.3389/frai.2023.1153083

**Published:** 2023-04-17

**Authors:** Daniel Cook, Matthew Biancalana, Nicole Liadis, Dorys Lopez Ramos, Yuhan Zhang, Snehal Patel, Joseph R. Peterson, John R. Pfeiffer, John A. Cole, Anuja K. Antony

**Affiliations:** SimBioSys, Inc., Chicago, IL, United States

**Keywords:** computational biomarker, ESBC, ICI, immuno-oncology, biophysical simulation, virtual tumors, virtual clinical trial, immune checkpoint inhibitor

## Abstract

**Background:**

Immuno-oncology (IO) therapies targeting the PD-1/PD-L1 axis, such as immune checkpoint inhibitor (ICI) antibodies, have emerged as promising treatments for early-stage breast cancer (ESBC). Despite immunotherapy's clinical significance, the number of benefiting patients remains small, and the therapy can prompt severe immune-related events. Current pathologic and transcriptomic predictions of IO response are limited in terms of accuracy and rely on single-site biopsies, which cannot fully account for tumor heterogeneity. In addition, transcriptomic analyses are costly and time-consuming. We therefore constructed a computational biomarker coupling biophysical simulations and artificial intelligence-based tissue segmentation of dynamic contrast-enhanced magnetic resonance imaging (DCE-MRIs), enabling IO response prediction across the entire tumor.

**Methods:**

By analyzing both single-cell and whole-tissue RNA-seq data from non-IO-treated ESBC patients, we associated gene expression levels of the PD-1/PD-L1 axis with local tumor biology. PD-L1 expression was then linked to biophysical features derived from DCE-MRIs to generate spatially- and temporally-resolved atlases (virtual tumors) of tumor biology, as well as the *TumorIO* biomarker of IO response. We quantified *TumorIO* within patient virtual tumors (*n* = 63) using integrative modeling to train and develop a corresponding *TumorIO Score*.

**Results:**

We validated the *TumorIO* biomarker and *TumorIO Score* in a small, independent cohort of IO-treated patients (*n* = 17) and correctly predicted pathologic complete response (pCR) in 15/17 individuals (88.2% accuracy), comprising 10/12 in triple negative breast cancer (TNBC) and 5/5 in HR+/HER2- tumors. We applied the *TumorIO Score* in a virtual clinical trial (*n* = 292) simulating ICI administration in an IO-naïve cohort that underwent standard chemotherapy. Using this approach, we predicted pCR rates of 67.1% for TNBC and 17.9% for HR+/HER2- tumors with addition of IO therapy; comparing favorably to empiric pCR rates derived from published trials utilizing ICI in both cancer subtypes.

**Conclusion:**

The *TumorIO* biomarker and *TumorIO Score* represent a next generation approach using integrative biophysical analysis to assess cancer responsiveness to immunotherapy. This computational biomarker performs as well as PD-L1 transcript levels in identifying a patient's likelihood of pCR following anti-PD-1 IO therapy. The *TumorIO* biomarker allows for rapid IO profiling of tumors and may confer high clinical decision impact to further enable personalized oncologic care.

## Introduction

Immunotherapy represents the leading edge in targeted therapeutics in early-stage breast cancer (ESBC), and immune checkpoint inhibitor (ICI) strategies are rapidly being integrated into the medical oncologist's repertoire of therapies (Gennari et al., 2021; Rizzo et al., 2022). Deployment of immuno-oncology (IO) treatment in ESBC is gaining momentum, with ICI antibodies targeting the PD-1/PD-L1 axis (namely pembrolizumab, nivolumab, and durvalumab) garnering substantial clinical attention and designation as the standard of care (SOC) within some cancer subtypes (Nanda et al., 2020; Schmid et al., 2020). Although ICI has proven to be a powerful therapeutic avenue, only 12.5% of patients overall successfully respond to IO (Haslam and Prasad, 2019; Haslam et al., 2020). As toxicity and adverse events from these potent IO agents are non-trivial, predicting patient response to immunotherapy is a pivotal next step in advancing precision IO therapies (Savas and Loi, 2020; Magbanua et al., 2022; Tarantino et al., 2022).

Determining a tumor's responsiveness to IO prior to initiating immunotherapy is a significant hurdle for precision IO implementation in ESBC. The cell surface protein programmed death ligand 1 (PD-L1) expressed on many cancer cells is a well-investigated biomarker of tumor responsiveness to ICI therapies targeting the PD-1/PD-L1 pathway. Frustratingly, currents methods to assess levels of PD-1/PD-L1 in ESBC are only marginally prognostic (Vranic et al., 2021; Zhao et al., 2022), highlighting the complexity of capturing the immune response and correlating it to the likelihood of IO success. Immunohistochemistry (IHC), although widely used to assess PD-L1 pathology, is nevertheless highly variable and dependent on experimental methodology (Patel and Kurzrock, 2015). An opportune moment therefore exists for employing innovative approaches incorporating mathematical modeling (Caballo et al., 2022; Howard et al., 2022), with the intent to develop novel biomarkers to determine tumor IO responsiveness, with a forthcoming need in ESBC (Franzoi et al., 2021).

To date, attempts at IO biomarker development have focused on single-site biopsy-derived tumor cell surface markers and tumor genetics. Use of gene microarrays (Bonsang-Kitzis et al., 2016) and next generation RNA sequencing (RNA-seq) to analyze the continuously changing cellular transcriptome has emerged to attempt to capture the dynamic patient immune response in ESBC (Iwase et al., 2021; Saltman et al., 2022; Seitz et al., 2022). Despite RNA-seq's enriched capacity, transcriptomic properties from a focal region may not accurately reflect the overall transcriptional behavior of the tumor, or the complexity of the dynamic immune tumor microenvironment (TME). Biopsy-driven approaches to tumor IO response characterization rely on single-site analyses or employ cell-line derived organoids (Scognamiglio et al., 2019). Both methods are fundamentally limited by the inherently heterogeneous nature of tumors, which feature regions bearing multiple genetic lineages. Additionally, such methods fail to accurately account for the influence of tumor spatial positioning, morphology, and blood supply.

Given these limitations, innovative approaches to biomarker development, which allow for spatial profiling of the entire tumor, are warranted. Ideally, such methods would be non-invasive, prognostic before IO therapy implementation, accurate in pathologic complete response (pCR) prediction, and rival current time-intensive and costly approaches. The pronounced lag in integrating both genomic and transcriptomic testing into the cancer treatment pipeline (Schilsky and Longo, 2022) argues for mathematical approaches that seamlessly combine SOC patient data and therapy response analysis.

**Figure 1:**
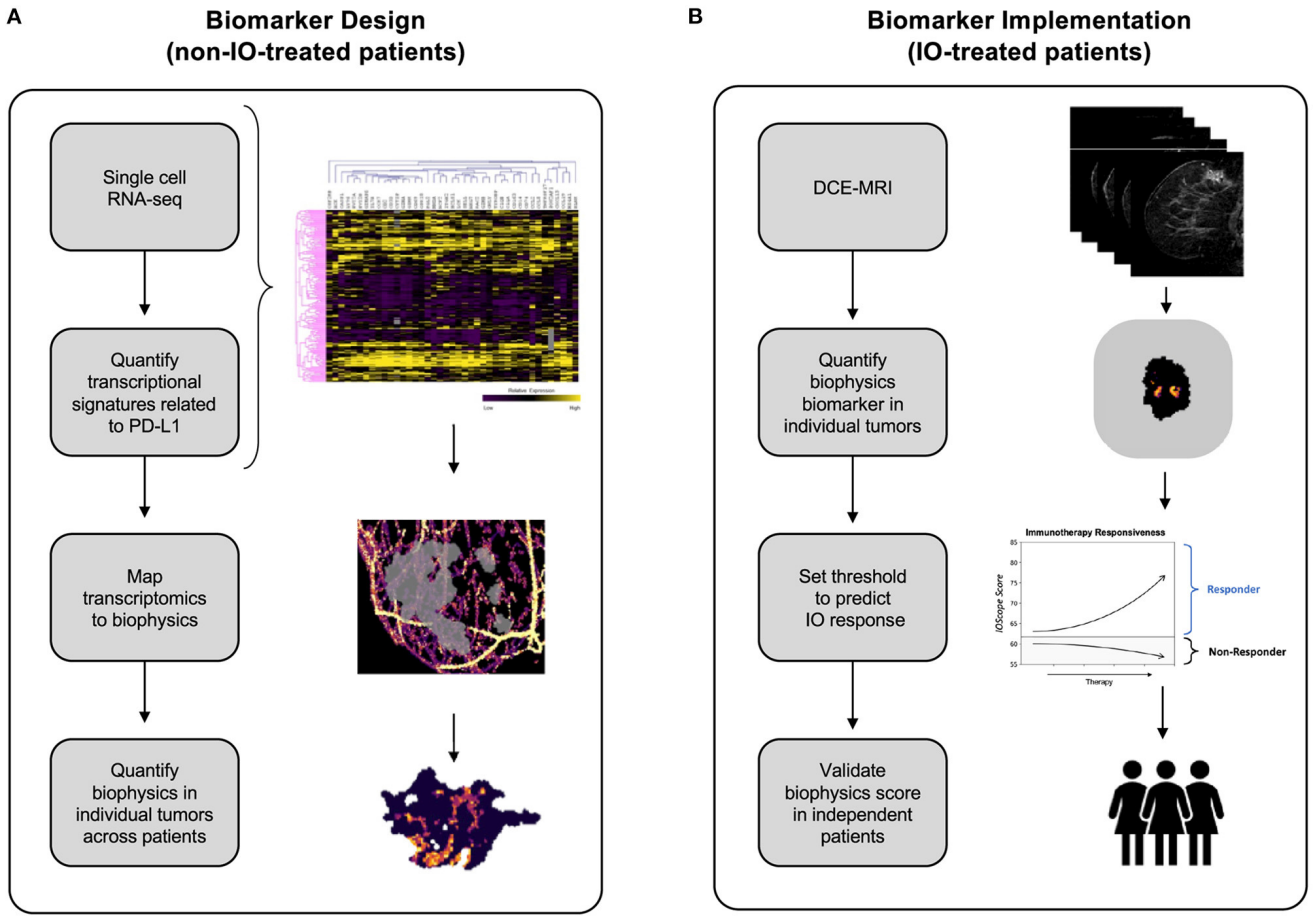
Development of the *TumorIO* biomarker, as well as the design and implementation of the *TumorIO Score* predicting response to IO therapy. **(A)** The *TumorIO* biomarker was developed using non-IO-treated (and pre-IO-treated) patients from the I-SPY1 and I-SPY2 clinical trials, as well as single cell RNA-sequencing data. Transcriptomic gene expression signatures of cancer hallmarks were used to quantify metabolic activity and angiogenesis within single cells. These signatures were then correlated with PD-L1 expression to quantify the relationship between PD-L1 expression and tumor metabolic activity and angiogenesis. Subsequently, metabolic activity and angiogenesis were quantified within patient tumors based on biophysical modeling of DCE-MRI image series to generate spatially resolved probability maps of PD-L1 expression within individual patient tumors. **(B)** The *TumorIO* biomarker was implemented using IO-treated patients from the I-SPY2 clinical trial to develop the *TumorIO Score* associated with likelihood of pCR in response to IO therapy. The *TumorIO Score* was then validated in an independent set of patients and through a virtual clinical trial.

We address these conventional biomarker pitfalls by employing and integrating validated biophysics-based computational biology methods (Howard et al., 2022) in order to predict IO therapy response. We have previously demonstrated that our technology's data predictions match with radiologist-verified assessment. The *Simul-omics 4D Engine* links dynamic contrast enhanced magnetic resonance imaging (DCE-MRI) to *in vivo* tumor behavior, thus alleviating the burden of information derived from a tissue-based biopsy. Simulations performed through the platform take <2 h to run, bypassing lengthy turnaround times inherent to other tissue-based methods. We describe an IO response biomarker termed *TumorIO* that is derived from an *in silico* training methodology, taking advantage of mathematical modeling strategies to characterize *in vivo* tumor activity (Figure 1).

We assembled two companion gene lists corresponding to essential tumorigenic features, reflecting angiogenesis and metabolic activity. Both of these processes are critical aspects of the “hallmarks of cancer” (Hanahan and Weinberg, 2000, 2011; Hanahan, 2022) and thus impact key tumorigenic pathways.

Using transcriptomic profiles derived from IO-naïve ESBC patients, we constructed biophysical signatures reflecting the underlying biological processes of these pathways. Virtual tumors recapitulating spatial biophysical features of angiogenesis and metabolic activity across time were then used to correlate PD-L1 transcriptional levels to spatial biophysical features derived from patient DCE-MRIs. Together, these features of the virtual tumors were used to generate the biophysics and imaging-dynamics-based *TumorIO* biomarker. We subsequently identify tumor-specific features that correlate with ICI response by training against publicly-available datasets containing paired transcriptomics and DCE-MRIs. We additionally computed a *TumorIO Score* to indicate the relative likelihood of pCR in response to ICI therapy. Together, the *TumorIO* biomarker and its corresponding *Score* reflect a next generation biophysical approach to accurately predict pCR to IO therapy.

## Methods

### Overview of study design

This analysis proceeded in two stages: (1). *TumorIO* biomarker design, and (2). *TumorIO Score* implementation.

In the design stage of the study, we analyzed publicly-available single-cell RNA sequencing (scRNA-seq) data from ER+ and triple negative breast cancer (TNBC) tumors to identify transcriptomics-based biological features associated with PD-L1 expression (Figure 1A). We used scRNA-seq data to quantify PD-L1 expression heterogeneity across individual tumors. We then correlated single cell PD-L1 expression with transcriptional biomarkers related to metabolic activity (Supplementary Figures 1, 2) and angiogenesis (Supplementary Figure 3). PD-L1 expression throughout the tumor cannot be visualized using SOC imaging; therefore, as a surrogate for spatially-resolved PD-L1 expression, we simulated and quantified the spatial distribution of both metabolic activity and angiogenesis within individual patient tumors and the TME using the *Simul-*omics *4D Engine* (Howard et al., 2022). This analysis resulted in a spatial map of biological features correlated with PD-L1 expression probability, which together underly the *TumorIO* spatial biomarker.

In the implementation stage of the study, we initially assessed processes enabling cancer cells to direct blood vessel generation and funnel local metabolic pathways to feeding tumor growth, both of which are core metabolic features of cancer establishment and progression. These “hallmarks of cancer” (Hanahan and Weinberg, 2000, 2011; Hanahan, 2022) impact essential tumorigenic pathways. We assembled two companion gene lists corresponding to these features, reflecting angiogenesis and metabolic activity. We initially quantified the spatial distributions of these two gene expression signatures within each tumor prior to IO therapy. We then computed summary features associated with these spatial maps and implemented a linear model on these summary features along with a threshold using a training data set to generate a *TumorIO Score* associated with pCR in response to IO therapy. We validated the *TumorIO Score* in an independent cohort of patients treated with IO and *via* a virtual clinical trial simulating IO administration (Figure 1B).

### Gene expression data retrieval and data normalization

Gene expression microarray data from breast cancer patients participating in the I-SPY1 (*n* = 221) and I-SPY2 (*n* = 972) clinical trials were downloaded from the gene expression omnibus (GEO, https://www.ncbi.nlm.nih.gov/geo; accession codes: GSE25055, GSE22226, GSE181574, GSE149322, GSE173839, GSE180962, and GSE194040).

For comparison with single cell data, microarray data were quantile-normalized to pseudo-TPM by scaling expression values for each gene to quantile-match RNA-seq data from the Cancer Genome Atlas Breast Cancer dataset (TCGA BRCA, https://www.cancer.gov/tcga; Koboldt et al., 2012). Pseudo-TPM was utilized solely for discovery to identify biologic features to be employed. Pseudo-TPM did not govern any of the results and was not incorporated into other aspects of the methodology. Linear regression was employed to avoid overfitting that may have resulted from alternative methods. Briefly, for each gene, log-transformed expression values were scaled such that the upper 90% quantile and the 0% quantile-matched the corresponding gene expression data from TCGA BRCA.

Datasets corresponding to scRNA-seq of ER+ and TNBC tumors were downloaded from the GEO (accession code: GSE161529). The data were analyzed without further normalization.

### Defining and calculating gene expression signatures with RNA-seq data

Metabolic activity and angiogenesis gene expression scores were calculated from curated gene expression signatures (Supplementary Figures 1–3). For each signature, the expression score was calculated as the weighted sum of the log-transformed expression levels of genes with positive weights minus the weighted sum of the log-transformed expression levels of genes with negative weights, as follows:


$$\begin{array}{l} Expression\;score=\;\frac{\sum_{w_{i}>0}\left(w_{i}E_{i}\right)}{\sum_{w_{i}>0}\left(w_{i}\right)}-\;\frac{\sum_{w_{i}<0}\left(w_{i}E_{i}\right)}{\sum_{w_{i}<0}\left(w_{i}\right)} \end{array}$$

where w_i_ is the weight of each gene in the expression signature and E_i_ is the expression level of the associated gene, in units of log_10_(TPM + 1). TPM is the transcripts per million reads derived from next generation sequencing (Zhao et al., 2020).

For the metabolic activity score, two individual scores were summed: the primary metabolic activity score and the secondary metabolic activity score. Supplementary Figures 1–3 present the signature genes and expression levels corresponding to primary metabolism, secondary metabolism, and angiogenesis, respectively.

Pairwise correlations between PD-L1 expression levels and (a) the metabolic activity signature or (b) the angiogenesis signatures across single cells were calculated using Pearson correlation.

### DCE-MRI imaging retrieval

Baseline DCE-MRIs for breast cancer patients participating in the I-SPY1 (*n* = 221) and I-SPY2 (*n* = 972) clinical trials were additionally downloaded from TCIA.

### Biophysical simulation of virtual tumors from DCE-MRI imaging series

**Figure 2:**
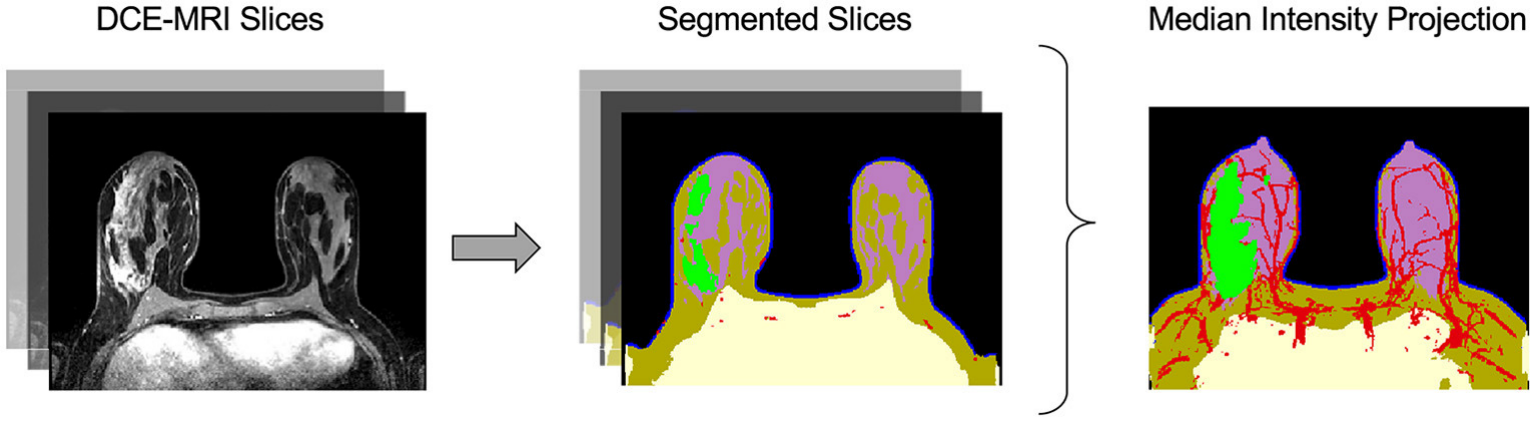
Automatic image segmentation of breast DCE-MRIs performed by artificial intelligence. The segmentation model uses a convolutional neural network (CNN) to identify and segment tissues based on multiple DCE-MRI slices **(left)**. The tissue categories of the segmentation are: air (black), chest (yellow), skin (blue), fat (brown), gland (purple), vasculature (red), and tumor (green), shown at center. The median intensity projection across all segmented layers is shown at **right**.

Virtual tumors were created from DCE-MRIs using the *Simul-omics 4D Engine* biophysical modeling platform (Howard et al., 2022). This platform uses artificial intelligence in the form of a convolutional neural network (CNN) to segment patient DCE-MRIs into regions corresponding to tumor, vasculature, and surrounding tissue automatically (followed by manual review; Figure 2). Following segmentation, the biophysical modeling platform uses these patient-specific virtual tumors coupled with biophysical analysis to calculate spatial maps of blood flow within and around a tumor, spatially-resolved delivery of nutrients to the tumor and surrounding tissue, and spatially-resolved atlases of tumor biology in 3D across the tumor volume. These atlases are then simulated forward in time to create spatially- and temporally-resolved predictions of tumor growth, tumor death in response to neoadjuvant chemotherapy, and subsequent likelihood of clinical pCR (Howard et al., 2022). The *Simul-omics 4D Engine* is ideal for designing spatial biomarkers because it allows for an individual's unique tumor biology to be simulated and interrogated in both space and time—an approach we have previously described and validated (predicting tumor response to neoadjuvant chemotherapy) with high rates of predictive accuracy (Howard et al., 2022).

### Developing a linear model to predict pathologic complete response in patients treated with chemotherapy plus immunotherapy

Biophysical simulations were performed on DCE-MRIs from I-SPY2 patients (*n* = 63) treated with pembrolizumab immunotherapy plus paclitaxel chemotherapy (see Table 1). The results of these simulations were used to compute metabolic activity and angiogenesis maps of each tumor and its TME. Summary features were then calculated from these maps.

**Table 1:** Patient characteristics for data used in this work.

	**Training**	**Validation**	**Virtual trial**
Age^*^	50 (27–71)	44 (32–69)	49 (23–85)
**Race**			
Caucasian	79.4%	23.5%	61.3%
African Am.	9.5%	11.8%	28.8%
Other	11.1%	64.7%	9.9%
**Subtype**			
HR+/HER2-	57.1%	29.4%	45.9%
TNBC	42.9%	70.6%	54.1%
**T-stage**			
T1	3.2%	0%	7.5%
T2	39.7%	52.9%	54.5%
T3	39.7%	35.3%	29.1%
T4	17.4%	0%	2.8%
Unknown	0%	35.3%	6.1%
Total no. of tumors	63	17	292

* Age is given as median (range).

For each cancer subtype (ER+ and TNBC), the summary features were then used to train a subtype-specific linear regression model that outputs a *TumorIO Score* associated with likelihood of pCR, according to the following equation:


$$\begin{array}{l} TumorIO\;Score=a_{1}\times \left(metabolic\;activity\right)+a_{2}\times \left(angiogenesis\right)+b \end{array}$$

where a_1_, a_2_, and b are trained parameters.

### Orthogonal validation in independent cohort

We validated the prognostic capability of the biophysical *TumorIO Score* in an independent cohort of patients (*n* = 17 of 141, University of Chicago Hospitals) who received an ICI-based immunotherapy regimen (pembrolizumab) in addition to SOC chemotherapy (paclitaxel) (see Table 1).

### Virtual clinical trial

A large cohort of patients (*n* = 292) were selected from the SimBioSys *Virtual TumorBank* (Howard et al., 2022), a databank of virtualized tumors (digital twins) generated using de-identified data from publicly-available datasets or private institution datasets under shared-use agreements (see Table 1). These data were used to perform a virtual clinical trial to assess the frequency with which *TumorIO* predicts immunotherapy response. The treatment arm of the trial was generated by calculating an *TumorIO Score* for each patient. The rates of pCR under IO treatment were then estimated by comparing the *TumorIO Score* for each patient to a threshold value calculated from the training dataset (I-SPY2 IO-treated patients). We used published trial data as the “control arm” for comparison.

### Statistical analyses

Computational model development, subsequent biomarker generation, and statistical analyses were performed in Python (Michel et al., 2011), Spyder (Raybaut, 2009), and R and R Studio (RStudio Team, 2020). Linear regression models were computed using the scikit learn package in Python (Michel et al., 2011).

Odds ratios of predicted response to IO plus chemotherapy were calculated as follows, with 95% confidence intervals calculated using the following formulas:

**Table:** 

	**Clinical pCR**	**Clinical residual disease**
Predicted pCR	TP	FP
Predicted residual disease	FN	TN

where TP is true positive, FP is false positive, FN is false negative, and TN is true negative. The odds ratio is therefore defined as:


$$\begin{array}{l} Odds\;ratio\;=\;\frac{TP\;\times \;TN}{FP\;\times \;FN} \end{array}$$

The following formulas were used to calculate a confidence interval (CI) for the odds ratio:


$$\begin{array}{l} Lower\;95\%\;CI\;=e^{\ln\left(OR\right)\;-\;1.96\sqrt{\left(TP^{-1}\;+\;FP^{-1}\;+\;FN^{-1}\;+\;TN^{-1}\right)}}\\ Upper\;95\%\;CI\;=e^{\ln\left(OR\right)\;+\;1.96\sqrt{\left(TP^{-1}\;+\;FP^{-1}\;+\;FN^{-1}\;+\;TN^{-1}\right)}} \end{array}$$

In the virtual clinical trial, response rates in the general population were calculated according to the equation below, with 95% confidence intervals calculated using the Clopper-Pearson exact method from the scikit-learn package in Python (Michel et al., 2011).


$$\begin{array}{l} Observed\;response\;rate\;=\frac{\#\;observed\;pCR}{total\;sample\;size}\\ Predicted\;response\;rate\;=\frac{\#\;predicted\;pCR}{total\;sample\;size} \end{array}$$

## Results

### Using gene expression analysis to identify biological features associated with PD-L1 expression

**Figure 3:**
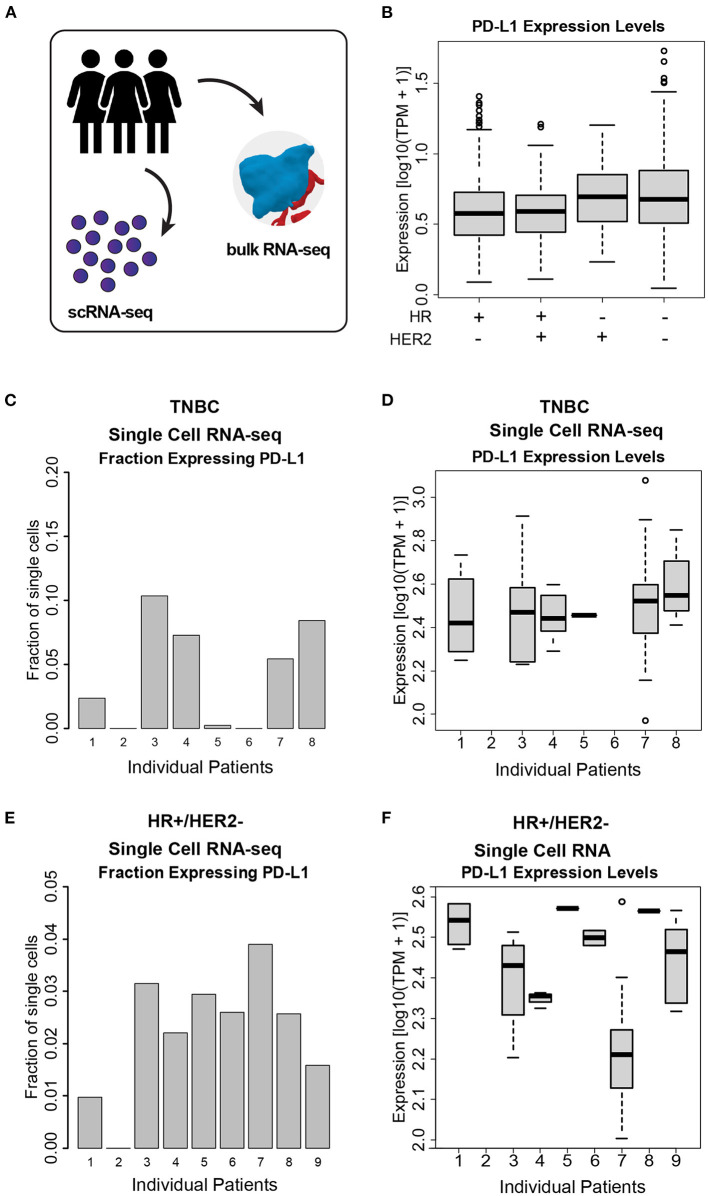
PD-L1 heterogeneity across and within ESBC tumors. **(A)** Schematic of experimental approach depicting use of both scRNA-seq and whole-tumor bulk RNA-seq. **(B)** PD-L1 expression levels in bulk RNA-seq from 972 ESBC patients from the I-SPY2 trial. **(C)** Fraction of cells expressing PD-L1 in TNBC tumor cells, as judged by scRNA-seq from eight patients. **(D)** PD-L1 expression levels within single cells across TNBC tumors. **(E)** Fraction of cells expressing PD-L1 in HR+/HER2- tumors, as judged by scRNA-seq from nine patients. **(F)** PD-L1 expression levels across HR+/HER2- tumors. In **(C–F)**, individual patients with no representative data indicate that no PD-L1+ cells were detected in these samples (i.e., HR+/HER2- patient 2, TNBC patient 2, and TNBC patient 6).

We quantified PD-L1 transcript level in bulk RNA-seq data across a total of 972 patients from the I-SPY2 trial, and we observed a wide range of PD-L1 expression both within and across cancer subtypes (Figures 3A, B). We additionally used transcriptomic data from single breast cancer cells (scRNA-seq) to quantify PDL1 expression across single cells within individual tumors (Figure 3A). We found that the fraction of cells within tumors expressing PD-L1 (Figures 3C, E) and the expression level of PD-L1 in the individual cells expressing PD-L1 (Figures 3D, F) are both highly variable. For instance, within a single patient, 40% of cells express PD-L1 with detectable levels (TPM > 1), and were therefore considered to be positive for PD-L1. Of those cells, the expression level of PD-L1 varied by >2 orders of magnitude. This degree of wide variability was apparent in both HR+ and TNBC tumors.

**Figure 4:**
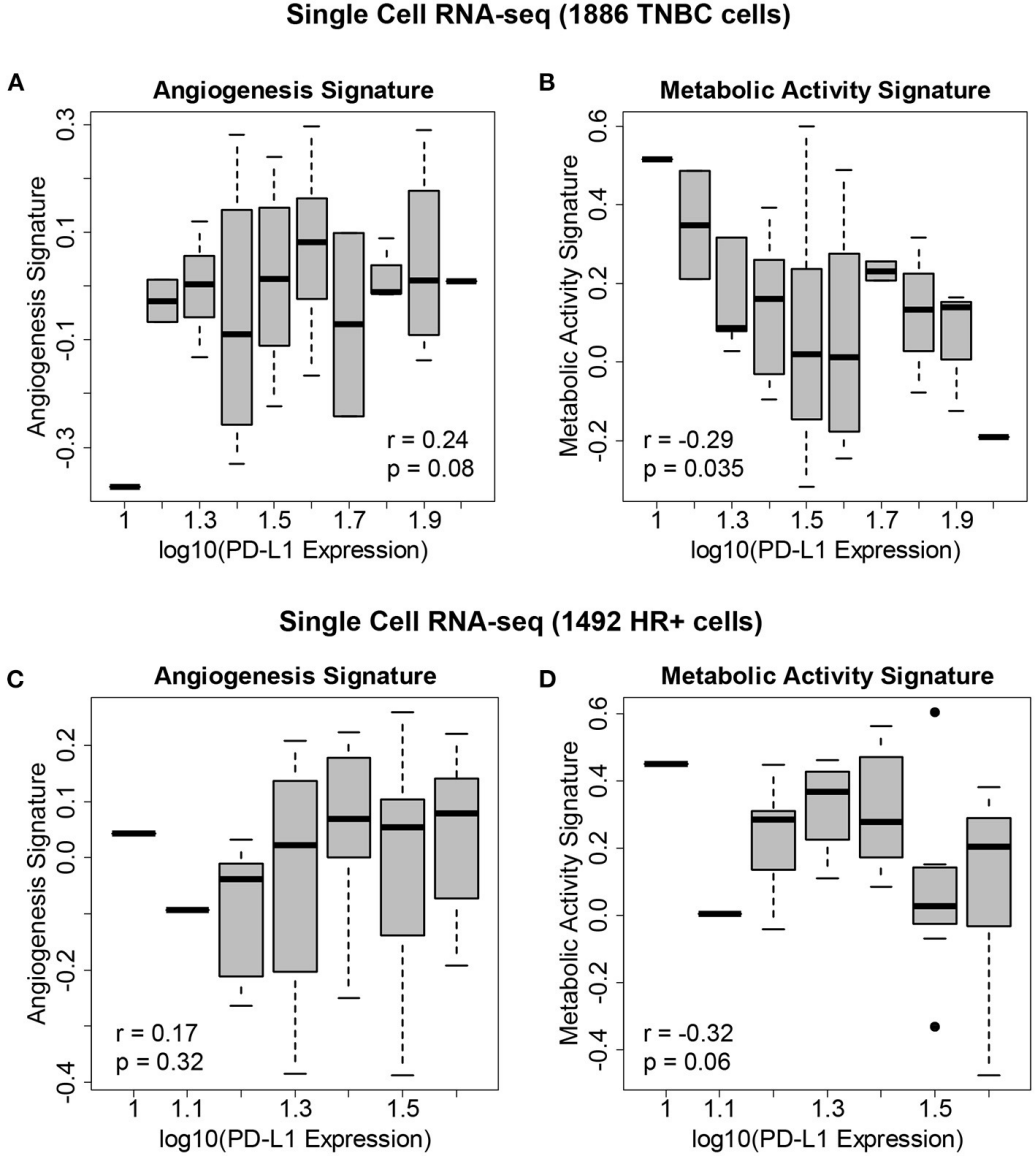
Gene expression signatures associated with PD-L1 expression in single breast cancer cells. **(A)** Levels of the angiogenesis signature across 1,886 cells derived from eight TNBC patients vs. PD-L1 expression, showing an association between angiogenesis and PD-L1 levels. **(B)** Levels of the metabolic activity signature across these TNBC cells vs. PD-L1 expression, showing an association between metabolic activity and PD-L1 levels. **(C)** Levels of the angiogenesis signature across 1,492 cells derived from 9 HR+/HER2- patients vs. PD-L1 expression. **(D)** Levels of the metabolic activity signature across these HR+/HER2- cells vs. PD-L1 expression.

We hypothesized that the state of the TME was associated with PD-L1 expression. We therefore correlated PD-L1 transcript levels across single cells with gene expression signatures of metabolic activity and angiogenesis (Figure 4). Within both TNBC tumors (Figures 4A, B—reflecting 1,886 TNBC cells derived from eight patients) and ER+ tumors (Figures 4C, D—reflecting 1,492 cells derived from nine patients), we found that PD-L1 expression was negatively correlated with metabolic activity and positively correlated with angiogenesis. We assessed the correlation between PD-L1 expression and the presence of these features using single cell RNA-seq. The following correlations with PD-L1 in TNBC cells were determined: angiogenesis (*r* = 0.24, *p* = 0.08) and metabolic activity (*r* = −0.29, *p* = 0.035). The following correlations with PD-L1 in HR+ cells were determined: angiogenesis (*r* = 0.17, *p* = 0.32) and metabolic activity (*r* = −0.32, *p* = 0.06). Although these correlations were modest, they were deemed of sufficient utility to warrant further investigation.

### Designing a biophysical signature associated with PD-L1 expression probability

The correlation of the metabolic activity and angiogenesis gene expression signatures with PD-L1 expression provide insight into localized tumor biology, but these data do not address the challenge of quantifying intra-tumor PD-L1 heterogeneity resulting from distinct TMEs. We therefore designed biophysical signatures of PD-L1 expression in TNBC and HR+/HER2- tumors that can be used to create a probability map of PD-L1 expression from DCE-MRI imaging data within and across individual patient tumors.

We performed *in silico* simulations of 63 patients tumors treated with ICI plus chemotherapy using the *Simul-omics 4D Engine* (for details, see Methods). These simulations allow for *in silico* characterization of each patients' tumor in 3D, including characterization of metabolism, blood flow, nutrient delivery, physical forces in and around the tumor, and tumor cell growth rate. We took advantage of the finding that PD-L1 correlates with signatures of metabolic activity and angiogenesis at a single-cell scale (Figure 4) when designing our biophysics-based PD-L1 expression probability maps, as follows: Within our biophysical simulations, we identified a signature of metabolic activity related to *in vivo* glucose use by the tumor that directly influences tumor growth behavior. The *Simul-omics 4D Engine* can assess this glucose use by the virtual tumor at MRI resolution using a combination of reaction-diffusion kinetics and systems biology models. Areas of the tumor with low glucose use (below a specific threshold) lead to a predominantly slow-growing or non-growing phenotype, while areas of the tumor with high glucose use (above a threshold) lead to a predominantly rapidly proliferating phenotype. We therefore designed a spatially-resolved, biophysics-based tumor metabolic activity signature in HR+ and TNBC tumors that is zero for all cells with glucose use below threshold, allowing for significant growth that can be modulated by changes in nutrient availability.

We next developed a biophysics-based spatial signature of angiogenesis within patient tumors. As opposed to the metabolic activity signature that interrogated the tumor directly, we investigated angiogenesis in the TME by examining the vasculature in the localized area surrounding the tumor. Some of the first steps in angiogenesis involve weakening of the blood vessel walls, migration of endothelial cells, and a corresponding increase in vascular leakiness (Eelen et al., 2020). Vascular leakiness is simulated directly by the *Simul-omics 4D Engine*, and we therefore used the *Simul-omics* platform to generate maps of vascular leakiness within blood vessels surrounding the tumor.

**Figure 5:**
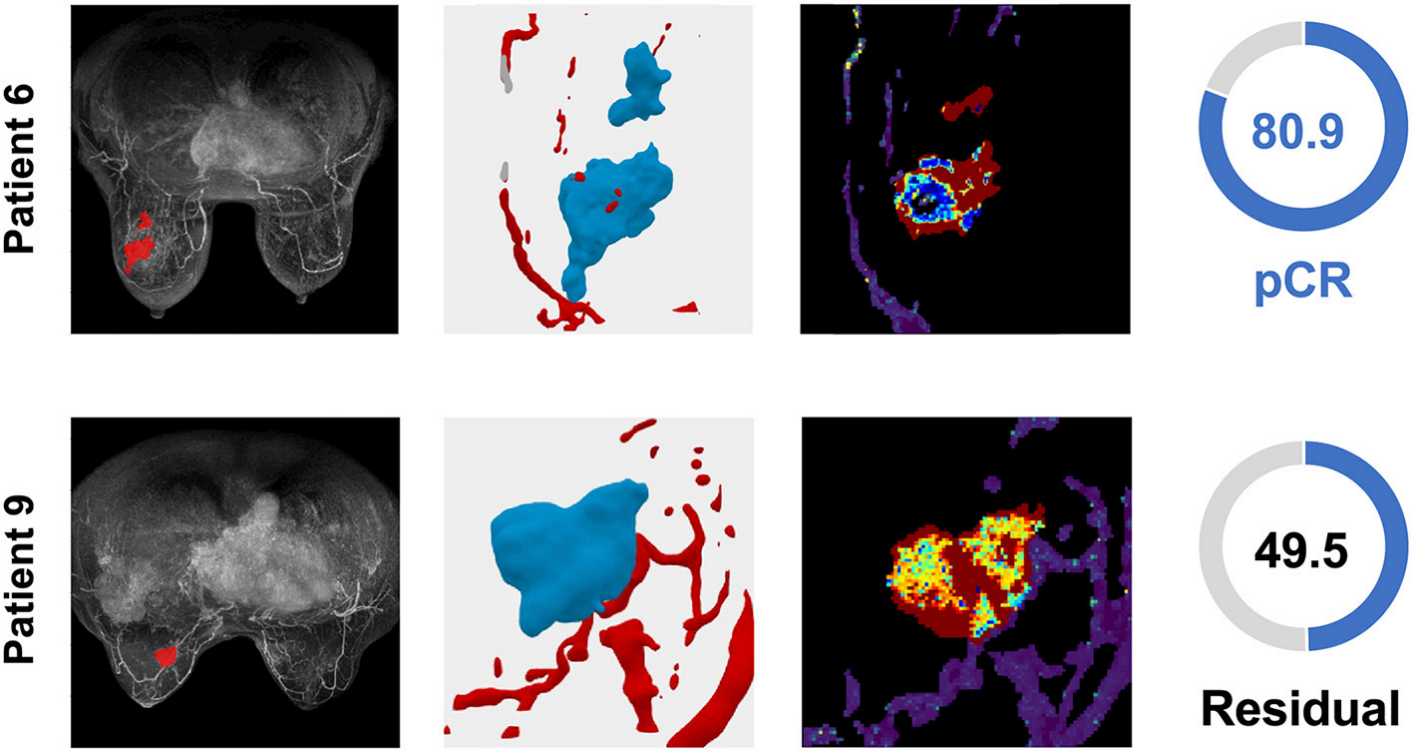
TNBC patient tumors from the validation cohort demonstrate the distribution of the *TumorIO* biomarker and utility of the *TumorIO Score* in predicting pCR in response to ICI therapy. The general workflow entails analysis of patient DCE-MRIs in order to generate virtual tumors. Spatial maps of *TumorIO* (reflecting metabolic activity and angiogenesis) show areas of the tumor and TME contributing to the *TumorIO Score*. The DCE-MRI dataset and resulting virtualized tumor are shown **(left** and **middle-left)**. The heatmap **(middle-right)** ranges from red to blue, representing a high to low gradient of the *TumorIO* biomarker within each tumor. These features are inversely correlated to the IO sensitivity of a tumor. In patient 6, low metabolic activity and areas of high angiogenesis contribute to a high *TumorIO Score* **(right)** and a correct prediction of pCR. In contrast, in patient 9, high metabolic activity and relatively few areas of angiogenesis contribute to a low *TumorIO Score* and a correct prediction of residual disease **(right)**.

Taken together, this spatial probability mapping can be thought of as taking the prognostic potential of a PD-L1 biomarker from a single biopsy site and expanding it to cover the entire tumor volume (a probability-based spatial map of biophysical features associated with PD-L1 biology; Figure 5, middle-right). We designed summary features based on the spatial variability of these maps for use in prognostic modeling. We used the spatially-resolved metabolic activity map to calculate tumor-wide biophysical metabolic activity by calculating the fraction of the tumor with spatial metabolic activity >0 (i.e., the fraction of the tumor with enough nutrients to support cell growth). In practice, this metabolic activity signature is directly related to the Metabolic Tumor Volume (MTV) commonly measured in PET scans, which reflects the metabolically active volume of the tumor.

For angiogenesis, 3D vascular leakiness maps were used to determine the fraction of vasculature in the TME actively involved in angiogenesis. We calculated the fraction of vasculature within the TME with leakiness greater 90% of the maximal possible leakiness (estimated across a sampling of patients from the I-SPY2 dataset). This fraction gives the relative amount of vasculature directly involved in angiogenesis surrounding each patient's tumor.

### Developing a TumorIO Score based on biophysics to predict pathologic complete response following immunotherapy

**Figure 6:**
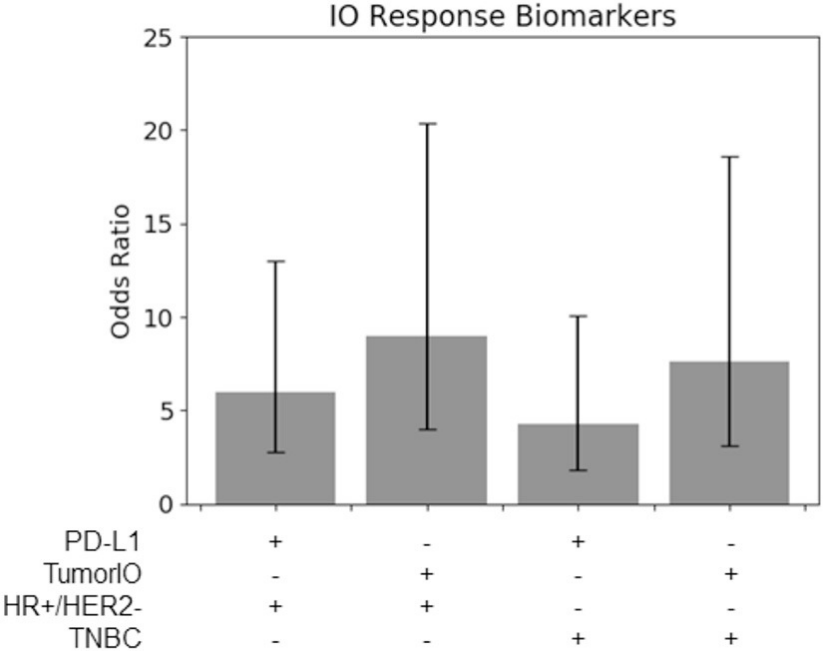
*TumorIO* analysis shows comparable efficacy to PD-L1 transcriptomics. Odds ratios of PD-L1 transcriptional levels, in comparison to the *TumorIO Score*, when predicting pCR in response to ICI therapy.

We used publicly-available DCE-MRIs of patients treated with immunotherapy (pembrolizumab) and chemotherapy (paclitaxel) from the I-SPY2 trial (*n* = 63) and the biophysical summary features described above (metabolic activity and angiogenesis) to train a linear regression model predicting an *TumorIO Score* associated with a patient's likelihood of pCR in response to IO therapy. We selected a threshold based on these 63 patients to predict pCR as a binary readout (either pCR or residual disease) to better compare with clinical practice (for example, with an odds ratio, see Figure 6).

### Comparing the prognostic power the *TumorIO Score* to transcriptomic-based PD-L1 expression

Using the 63 patients treated with IO in the I-SPY2 clinical trial, we found that for both TNBC tumors and HR+/HER2- tumors, the *TumorIO Score* was able to predict pCR with the same level of prognostic value as PD-L1 expression, as determined from transcriptomic analysis (overlapping 95% confidence intervals; Figure 6). Although these data hint at a potentially stronger prognostic power of the *TumorIO Score* over PD-L1, the small number of patients in this cohort make potential differences statistically indiscernible. Further analysis with larger patient cohorts will therefore be needed to refine this analysis.

### Validating the biophysical immune score in an independent patient population

We further validated the prognostic capability of the biophysical *TumorIO Score* in an independent population of patients (*n* = 141 total patients in cohort) from the University of Chicago Hospitals. Of these 141, 17 patients received an immunotherapy regimen (pembrolizumab) in addition to SOC chemotherapy (paclitaxel). The biophysical *TumorIO Score* correctly predicted pCR in 88.2% of the validation set (10/12 in TNBC and 5/5 in HR+/HER2- tumors; see Table 2 for HR+/HER2- and Table 3 for TNBC patients).

**Table 2:** Prediction of pCR in HR+/HER2- tumors (5/5 correct).

	***TumorIO Score***	**pCR prediction**	**True pCR call**
Patient 1	0.401	pCR	pCR
Patient 2	−1.060	Residual	Residual
Patient 3	0.713	pCR	pCR
Patient 4	0.255	Residual	Residual
Patient 5	−0.139	Residual	Residual

**Table 3:** Prediction of pCR in TNBC tumors (10/12 correct).

	***TumorIO Score***	**pCR prediction**	**True pCR call**
Patient 1	0.625	pCR	pCR
Patient 2	0.621	pCR	pCR
Patient 3	1.001	pCR	pCR
Patient 4	0.681	pCR	Residual
Patient 5	0.996	pCR	pCR
Patient 6	0.809	pCR	pCR
Patient 7	0.727	pCR	pCR
Patient 8	1.085	pCR	pCR
Patient 9	0.495	Residual	Residual
Patient 10	0.496	Residual	Residual
Patient 11	0.491	Residual	pCR
Patient 12	0.661	pCR	pCR

### Performing secondary validation in a virtual clinical trial

Given IO's recent presence in clinical trials, limited long-term data for IO-treated ESBC patients is available, and trial sponsors have been reluctant to share data on patient responses to ICI. Therefore, we performed a novel method of secondary validation by executing a virtual clinical trial using a large cohort of immunotherapy-naïve HR+/HER2- and TNBC patients (*n* = 292 virtualized tumors originating from multiple institutions) using the SimBioSys *Virtual TumorBank* (Howard et al., 2022). After simulating IO administration and determining IO response using the *TumorIO Score*, we predicted pCR rates of 67.1% for TNBC and 17.9% for HR+/HER2- tumors with the addition of IO therapy to a standard chemotherapy background (Table 4).

**Table 4:** Virtual clinical trial results using the *Virtual TumorBank*. Known pCR rates from chemotherapy alone vs. predicted pCR rates from chemotherapy plus pembrolizumab therapy, demonstrating an increase in response produced by immunotherapy.

	**Number of tumors**	**Chemo pCR rate**	**Chemo + IO pCR rate**
HR+/HER2-	134	9.0% (4.7–15.1)	17.9% (11.8–25.5)
TNBC	158	38.6% (31.0–46.7)	67.1% (59.2–74.3)

## Discussion

We demonstrate a novel method for biomarker development which integrates validated computational biology mathematical modeling strategies to design a biophysics-based signature of immunotherapy (ICI) responsiveness, specifically to blockade of the PD-1/PD-L1 axis. Predicated on dynamic imaging and perfusion kinetic-based features, this biophysics-based approach offers a powerful new strategy for non-invasively predicting tumor IO responsiveness and selecting patients for ICI therapy, conveying actionable guidance around predicted treatment response.

Utilizing the *TumorIO Score*, we correctly predicted pCR in 15 of 17 individuals in the independent validation cohort (88.2% accuracy, comprising 10/12 in TNBC and 5/5 in HR+/HER2-). Although promising, given the small sample size of our independent cohort, we developed an additional method to orthogonally validate the *TumorIO* biomarker utilizing a virtual clinical trial. Here, IO administration was simulated in an IO-naïve cohort, and pCR rates from the virtual trial were compared with published trials. Our virtual clinical trial approach predicted pCR rates of 67.1% for TNBC and 17.9% for HR+/HER2- tumors for IO therapy in the presence of a standard chemotherapy background. These predicted virtual trial pCR rates compare favorably to published rates from clinical trials utilizing IO in both cancer subtypes. The pCR rate in the I-SPY2 trial with pembrolizumab and SOC chemotherapy was 60% in TNBC and 30% in HR+/HER2- cancers (Nanda et al., 2020), while the GIADA trial demonstrated a pCR rate of 16.3% in IO-treated HR+/HER2- patients (Franzoi et al., 2021; Dieci et al., 2022). The success of this validation strategy supports the potential for expansion of virtual clinical trials in both research and drug development applications (Howard et al., 2022). Additionally, this result signals the potential of the *TumorIO Score* to accurately predict ICI responsiveness and provide a strong correlation to randomized control trials.

The *TumorIO* biomarker's composition of biophysical traits associated with angiogenesis and metabolic activity highlights that the nutrient microenvironment of a tumor is closely related to its response to ICI therapy. There is precedence for our findings that a tumor's ability to access and utilize nutrients is correlated (inversely) with IO response. For instance, 18F-fluorodeoxyglucose positron emission tomography/computed tomography (F-FDG PET/CT) data provide direct insight in metabolic activity of tumors. The FDG glucose-based tracer non-invasively measures glycolytic metabolism that presents a “metabolic signature” reflecting a tumor's overall energy consumption, in a conceptually similar manner to *TumorIO*. Recent F-FDG PET/CT studies suggest a negative correlation between baseline metabolic activity and ICI monotherapy response (Hindié, 2020; Lang et al., 2020), although the underlying etiology for this finding has been elusive in the radiology literature. Importantly, these data closely parallel our finding of low nutrient availability in tumors with strong ICI response. This correspondence suggests that the biology underlying the association between low metabolic activity and ICI responsiveness is fundamentally linked: nutrient deficits lead to high tumor PD-L1 spatial coverage across the tumor volume and, therefore, a priming of the TME for ICI responsiveness.

Our spatial simulations assess glucose delivery and uptake indirectly, using a combination of reaction/diffusion equations, pharmacokinetic/pharmacodynamic (PK/PD) modeling, and tumor morphology modeling, to characterize glucose delivery within the tumor and the tumor's resulting biological responses. This approach is analogous to the ability of F-FDG PET/CT to quantify glucose delivery to and uptake by the tumor, indicating the metabolically active regions of the tumor. Low metabolic tumor volume (MTV) values are associated with better response to ICI in lung cancer (Liao et al., 2022), bladder cancer (Girard et al., 2020), and metastatic melanoma (Flaus et al., 2021). Our data demonstrate a similar association in breast cancer, whereby low MTV, and therefore high *TumorIO Score*, is associated with strong response to immune checkpoint blockade.

**Figure 7:**
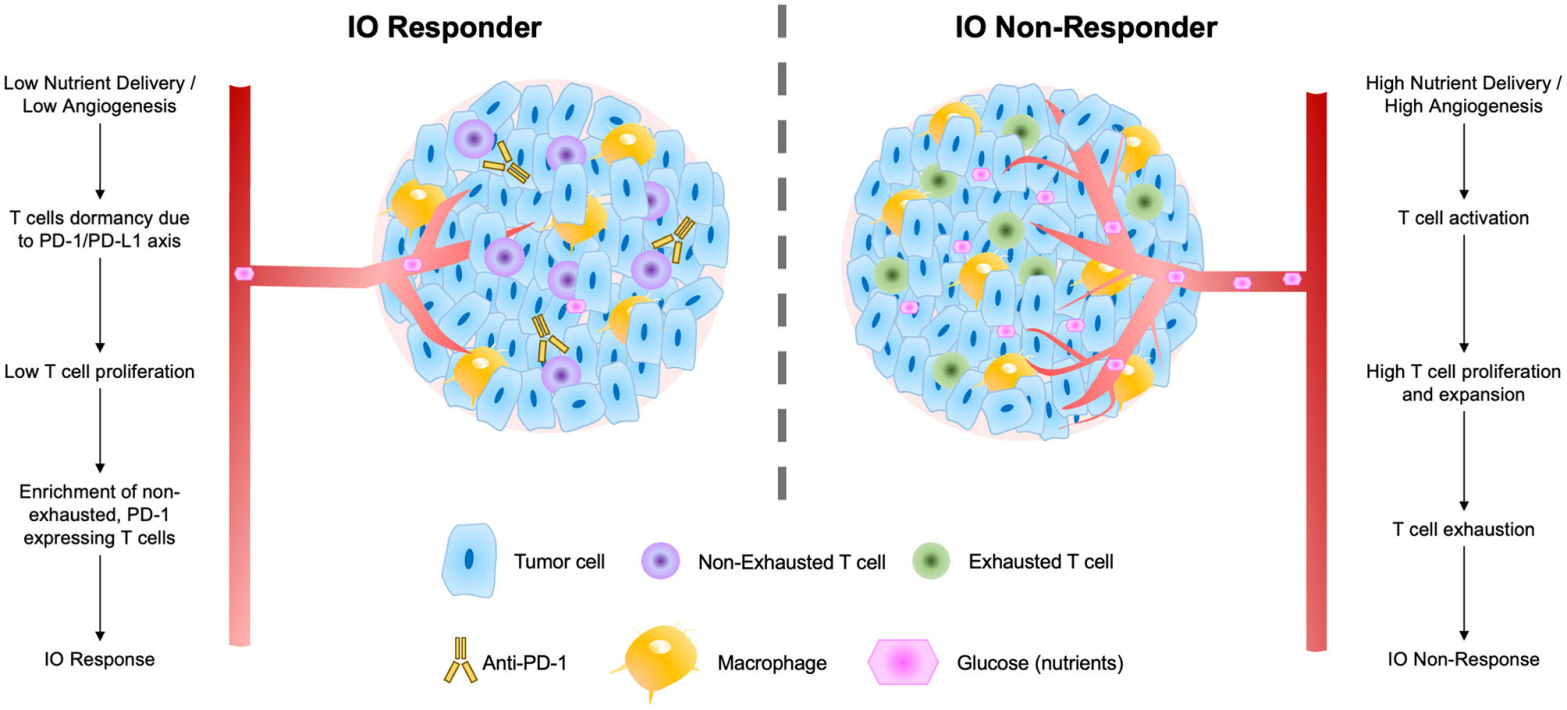
Schematic of factors contributing to tumor IO-sensitivity **(left)** and IO-insensitivity **(right)** both within tumors and within the local tumor microenvironment. These factors include degrees of nutrient delivery, T cell dormancy, T cell proliferation, and levels of exhaustion in local T cell populations.

These findings suggest a biological mechanism for response to ICI therapies that fits well within the larger body of T cell immuno-biology (Figure 7). In response to low nutrient availability (i.e., glucose), tumors up-regulate immune evasion genes (including PD-L1; Cha et al., 2019). Tumor infiltrating lymphocytes (TILs) and resident memory T cells (TRMs) in these regions are thereby inhibited (and remain non-exhausted) by the local microenvironment of the tumor. The presence of TRMs, particularly, is a strong predictor of IO response (Loi et al., 2014, 2019, 2022), and under low nutrient availability, these immune cells remain primed for potential activation *via* a co-stimulatory signal. When inhibitory interactions (e.g., PD-1/PD-L1) are removed through use of ICI therapies, these non-exhausted, activated T cells rapidly expand, producing an immune response facilitating elimination of the tumor.

Visualization of the *TumorIO* biomarker in 3D allowed us to identify local “hot spots” in IO-responsive tumors that corresponded with strong IO response (Figure 4, middle-right). Our biophysical maps of PD-L1 probability across the tumor volume indicate that the entirety of a tumor does not need to be PD-L1-high (or otherwise immune-responsive) to produce pCR. Instead, if a threshold mass of tumor is sensitive to IO, the whole tumor can be killed effectively. We hypothesize that the “hot spots” on these spatial maps of IO responsiveness may correspond to biological features intimately connected to the immune response to ICI. Previous research has implicated tertiary lymphoid structures (TLS) as critical organizing sites for immunotherapy response (Cabrita et al., 2020; Helmink et al., 2020; Vanhersecke et al., 2021; Italiano et al., 2022), featuring high immune cell density and a particular abundance of B cells in addition to cytotoxic T cells (Goc et al., 2014; Petitprez et al., 2020). The relative number of TLS within a tumor and/or its local microenvironment correlates with enhanced tumor death and improved patient survival (Sautès-Fridman et al., 2019). These computational biophysical maps may reflect these or related structural linkages between critical sites of immune cell density and functional immune response that lead to improved patient outcomes.

We present an overarching flow diagram connecting the observed features of IO-responsive tumors, as well as tumors that are resistant to IO therapy (Figure 7). This IO response model unifies many observed features of IO-responding and non-responding tumors. The model argues that tumors with slower overall growth rates and with an abundance of non-exhausted, PD-1-expressing T cells are most likely to respond to IO therapy (Baldominos et al., 2022). In fast growing tumors or those with limited T-cell infiltration, we speculate that treatment with anti-angiogenesis (anti-VEGF) agents may serve to prime these tumors to IO therapy and enhance IO responsiveness (Zalcman et al., 2016; Boucher et al., 2021; Fennell et al., 2022).

Next generation biomarkers derived from computational modeling offer a sophisticated, technology-based method to select patients best suited to IO therapy and afford insights into the underlying biologic drivers of tumor resistance and sensitivity to therapy. In the absence of discerning biomarkers, the healthcare cost burden due to unmitigated ICI usage is considerable. Targeting the PD-1/PD-L1 axis carries numerous secondary effects, particularly the potential for immune-related adverse events including sepsis, pneumonitis, and need for hospitalization. As a large subset of patients are unlikely to benefit from ICI, the morbidity-related costs to the healthcare system compound the already costly nature of IO.

The prognostic power of the biophysical simulation approach presented in this study highlights the capabilities of patient-specific biophysics-based signatures in predicting pCR and defining optimal therapy courses. The utility of these signatures, however, extends far beyond immunotherapies. Because these simulations are predicated on mathematical descriptions of tumor biology, they can be used to interrogate fundamental biological relationships within tumors and to predict individual patient responses to both SOC and novel regimens.

## Limitations

The primary limitation in this study is the small number of patients available for orthogonal validation in an independent cohort; thus, an additional validation strategy encompassing a virtual clinical trial was included. Despite immunotherapy becoming SOC in ESBC, alongside an ever-expanding cohort of IO therapies, there is a dearth of publicly-available empirical data. It is possible that with incorporation of additional patient datasets, accuracy may vary, and we are actively seeking additional independent datasets incorporating IO therapy to further validate the biomarker. In future, we anticipate further efforts will be needed on the part of our group and others for protocol standardization and validation efforts for expansion of next generation immuno-oncology tumor profiling into the clinical setting. Additionally, we have begun preliminary studies to explore how more comprehensive profiling of tumor behavior could lead to enhanced prognostic power. Data from more IO trials, however, will be necessary to fully validate these IO response signatures.

## Conclusions

We present a novel method of biomarker development predicated on biophysics-based modeling and validate its use, advancing personalized medicine initiatives. This strategy offers compelling advantages to traditional IHC assays as a non-invasive method of assessing IO-sensitivity. The *TumorIO Score* overcomes many of the limitations caused by the invasive, costly, and time-consuming nature of transcriptomic data acquisition and inability of single-site biopsies to account for tumor heterogeneity or the complexity of the immune response to ICI. Although further validation would be needed prior to implementation of this biomarker in clinical practice. Extensions of this underlying technology include future biomarker development in emerging oncology research areas, development of novel pharmaceuticals, as well as further investigations of underlying biological interactions responsible for tumor growth and destruction.

## Data availability statement

The original datasets presented in this study are available through a shared-use agreement with the partnering institution and have not yet been made available for public distribution. Requests to access the datasets should be directed to DC, daniel@simbiosys.com. The existing datasets analyzed as part of the study can be found in online repositories. The names of the repository/repositories and accession number(s) can be found in the article/Supplementary material.

## Ethics statement

Ethical approval was not required for the study involving human data in accordance with the local legislation and institutional requirements. Written informed consent to participate in this study was not required in accordance with the national legislation and the institutional requirements.

## Author contributions

DC, NL, YZ, SP, JPe, JPf, JC, and AA: conceptualization. DC, YZ, SP, JPe, JPf, JC, and AA: methodology. DC: validation, formal analysis, and investigation. NL, YZ, SP, JPe, JPf, JC, and AA: resources. DC, MB, and AA: writing—original draft preparation. DC, MB, DL, SP, and AA: writing—review and editing. DC, MB, and DL: visualization. SP, JPe, JPf, JC, and AA: supervision. AA: acts as the guarantor of the study. All authors have read and agreed to the published version of the manuscript, confirm that they had full access to all the data in the study, and accept responsibility to submit for publication.
