# Supplementary material for: Next generation immuno-oncology tumor profiling using a rapid, non-invasive, computational biophysics biomarker in early-stage breast cancer
Source: Front Artif Intell. 2023 Apr 17;6:1153083. doi: 10.3389/frai.2023.1153083 (PMC10149754; doi:10.3389/frai.2023.1153083)
Supplement: Supplementary file 1 [file Data_Sheet_1.PDF]

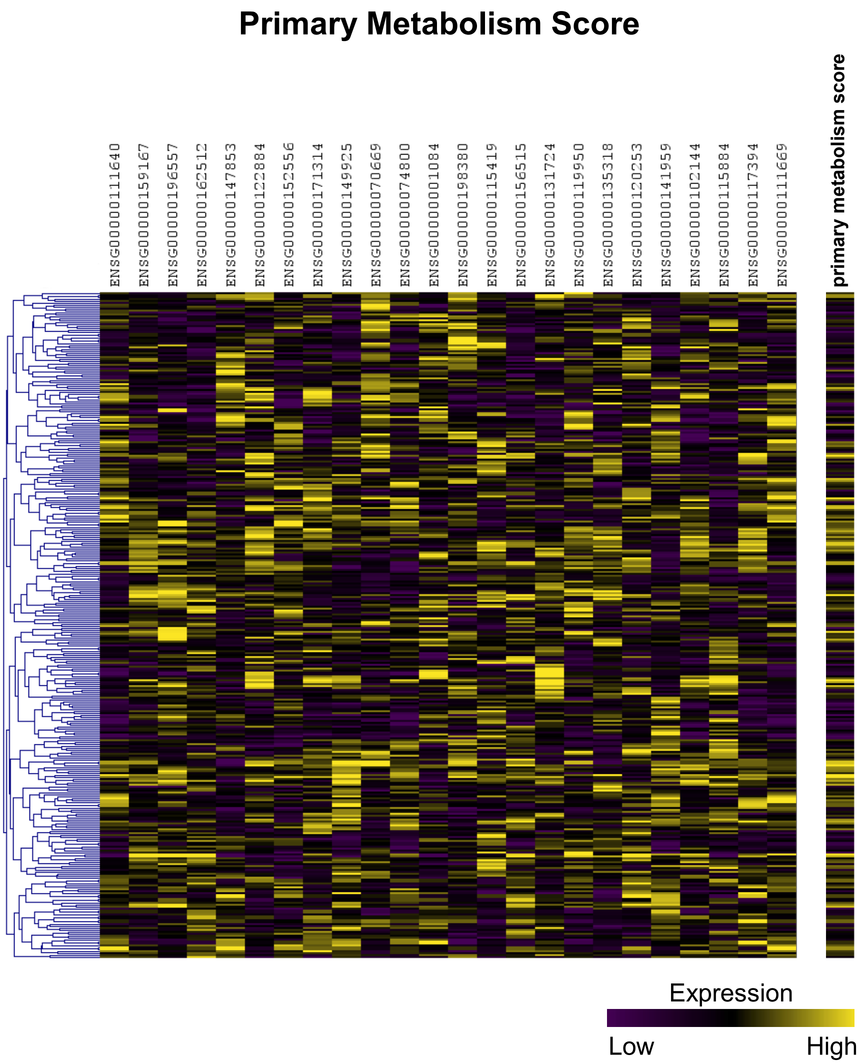

Figure S1. Signature genes and weights corresponding to primary metabolism, as reflected across 345 I-SPY2 patients. The resulting primary metabolism score (per patient) is shown in the right-most column. The data are derived from bulk RNA-seq analysis.
